# Seroprevalence of Old World Hantaviruses and Crimean Congo Hemorrhagic Fever Viruses in Human Populations in Northwestern Ukraine

**DOI:** 10.3389/fcimb.2020.589464

**Published:** 2020-10-22

**Authors:** Ihor Lozynskyi, Anna Shulgan, Olha Zarichna, Iryna Ben, William Kessler, Xueyuan Cao, Olena Nesterova, Gregory E. Glass, Briana Spruill-Harrell, Mariah K. Taylor, Evan P. Williams, Colleen B. Jonsson

**Affiliations:** ^1^Research Institute of Epidemiology and Hygiene of Danylo Halytskyi, Lviv National Medical University, Lviv, Ukraine; ^2^Department of Geography, University of Florida, Gainesville, FL, United States; ^3^College of Nursing, The University of Tennessee Health Science Center, Memphis, TN, United States; ^4^Black and Veatch Special Projects Corp., Kyiv, Ukraine; ^5^Department of Microbiology, Immunology and Biochemistry, The University of Tennessee Health Science Center, Memphis, TN, United States

**Keywords:** hantaviruses, Crimean Congo hemorrhagic fever virus, Ukraine, human population survey, Puumala virus, PUUV, Dobrava-Belgrade virus, DOBV

## Abstract

In Ukraine, a retrospective review of clinical case reports by public health officials suggest that human cases of febrile illnesses associated with hemorrhage may be due to infections of Crimean-Congo hemorrhagic fever virus (CCHFV) and Old World hantaviruses. In a serosurvey of 966 healthy individuals in the Lviv Oblast, Ukraine, bordering Poland, we found that 1.6% showed cross-reactivity to hantaviral antigens by an immunofluorescence assay (IFA) and 1.7% of the study participants had antibodies cross-reactive to CCHFV by enzyme-linked immunosorbent assay (ELISA). Demographic variables and history of exposures obtained through questionnaires were assessed by logistic regression models for association with seroprevalence for both viruses with no significant risk factors found. Analysis of spatial distribution identified two clusters of samples positive for antibodies to both hantaviruses and CCHFV, which, however, were not statistically significant (p > 0.05). In general, the study results suggest that the population of the study area is exposed to hantaviruses and CCHFV. Further surveillance for respective pathogens in Ukraine is warranted and prospective surveillance of febrile patients with unidentified febrile illness.

## Introduction

Hemorrhagic fever viruses causing febrile illness include Rift Valley Fever virus, Lassa fever virus, Crimean-Congo hemorrhagic fever virus (CCHFV), and several Old World hantaviruses. Based on the presence of vectors or reservoirs for CCHFV and hantaviruses in Ukraine, public health officials suggest that human cases of febrile illnesses may be due to exposure to these pathogens. Hantaviruses are widely distributed in nature in mice, shrews, voles and bats (Jonsson et al., 2010b; Vaheri et al., 2013a; Vaheri et al., 2013b), but the primary viruses responsible for illness (hemorrhagic fever with renal syndrome [HFRS]) in humans are harbored by wild rodents. In Europe, hantaviruses associated with HFRS include *Puumala orthohantavirus* (PUUV) and *Dobrava-Belgrade orthohantavirus* (DOBV) strains (Heyman et al., 2011), which are carried by the bank vole (*Myodes glareolus*) and the striped field mouse (*Apodemus agrarius*) or the yellow-necked mouse (*Apodemus agrarius*), respectively. In the northwestern region of Ukraine, all three species are present. The primary vector for CCHFV is the ixodid tick, with the *Hyalomma* species serving as the principal transmitter in nature (Messina et al., 2015a; Messina et al., 2015b). Although systematic surveillance studies to identify CCHFV in Ukraine have not been conducted, it is likely that the virus is widely present in southern regions of the country. Indeed the virus was first characterized in Crimea in 1944 and the illness was originally called Crimean hemorrhagic fever (Carroll et al., 2010). Ukrainian public health officials have detected CCHFV antigen in *Ixodes* ticks in several Oblasts of Ukraine, including Zakarpattia and Lviv.

When transmitted to humans, rodent-harbored hantaviruses can cause serious disease ranging from proteinuria to pulmonary edema and frank hemorrhage (Jonsson et al., 2010b). There are three DOBV genotypes that cause disease in humans: Belgrade, Kurkino, and Sochi (Papa, 2012; Hofmann et al., 2014; Krautkramer et al., 2016; Meier et al., 2018). While there are many similarities in their clinical manifestations in humans, one of the main differences between the strains DOBV-Belgrade and DOBV-Kurkino is their virulence and, therefore, their severity and case-fatality rate in infected patients. Both cause HFRS, which include a cluster of symptoms with five phases following an initial incubation period of 2 to 4 weeks. The DOBV-Kurkino genotype case fatality rate has been described as ranging from 0.3% to 0.9%; whereas, DOBV-Belgrade has been shown to be more virulent, with a case fatality rate of 10% (range, 5–15%) or greater (Hofmann et al., 2014). In fact, the clinical severity and case fatality rate of DOBV infection in humans appears to be closely linked to genotype. The molecular basis of virulence of different subtypes has yet to be understood. PUUV causes a mild form of HFRS called nephropathia epidemica (Settergren, 2000; Huttunen et al., 2011), which is characterized by fever, abdominal pain and/or back pain and/or headache, and signs of renal involvement. While initial symptoms have a sudden onset and are often severe (fever, chills vomiting, headache, and abdominal pain), the remaining clinical course is usually mild and self-limiting.

CCHFV is transmitted to humans by ticks through tick-bites, squashing engorged ticks, or with direct contact with blood or tissues of viremic humans or animals (Whitehouse, 2004). Person to person transmission can occur through contact with blood or bodily fluids. The case fatality rate seen with CCHF is two to three times higher than that of the most virulent HFRS, with estimates ranging from 30% to 50%.

To explore the potential contributions to human disease caused by hemorrhagic fever viruses in Ukraine, we collected and screened sera from healthy persons for antibodies against antigens of CCHFV, PUUV, and DOBV. Our survey was focused on healthy persons living in Yavoriv rayon of Lviv Oblast located in the northwestern region of the country. We hypothesized that exposure to ticks carrying CCHFV would be due to travel or seasonal introduction of vectors from commerce. Second, we hypothesized that exposure to wild rodents carrying hantaviruses would occur through rural activities and lifestyle.

## Materials and Methods

### Collection of Human Sera Samples and Demographics

The study was conducted according to the approved master protocol and in compliance with the regulatory requirements applicable to Ukraine and under all applicable Food and Drug Administration (FDA) regulations, International Conference on Harmonization (ICH) guidelines, and the US Department of Defense (DoD) requirements. The US Defense Threat Reduction Agency (DTRA) Human Research Oversight Board (HROB) reviewed and approved this research study, which was conducted in compliance with U.S. Army Regulation AR 70-25. The protocol, informed consent document, and US Army Medical Research Institute of Infectious Diseases (USAMRIID) Institutional Review Board (IRB) approval complied with 32 CFR 219, DoD 3216.2, DTRA Directive 3216.01, and DTRA Instruction 3216.02. The tracking and study numbers were, CT-2009-07-Other and FY 08-32, respectively. Records and all regulatory documents associated with the study are stored in a secure location at the Research Institute of Epidemiology and Hygiene (RIEH) of Danylo Halytskyi, Lviv National Medical University (Lviv, Ukraine). No personal identifiers have been used in any publication or communication used to support this research study. Inclusion Criteria included: age ≥ 18 years old, presenting to the study site for blood donation or medical care of a non-infectious nature and willingness and ability to participate by providing informed consent. The Exclusion Criteria were: age <18 years old, If a hospital patient, and currently diagnosed with significant anemia, a bleeding disorder, or other condition that their medical care team feels would be negatively impacted by participation in the study and ff, in the opinion of the investigator, inclusion in the study may not be in the best interest of the potential volunteer.

Each healthy volunteer completed an interview in which a trained field team epidemiologist administered a questionnaire (Supplemental Data), documenting their prior clinical history and risk of exposures to the infections studied in this protocol. This assessment occurred after the informed consent process was completed. The amount of blood drawn per consented individual was up to 10 ml. No more than 4 phlebotomy attempts were made per person. Whole blood, drawn in serum separator tube(s), was centrifuged to separate the cellular components of the blood from the serum. The serum was placed in 1 mL plastic tube aliquots and aliquots were stored at the LRIEH at −80°C.

### ELISA for CCHFV

We screened human sera with the Human CCHFV IgG ELISA Kit (Abbexa Ltd., Cambridge Science Park). The reagent kit tests for reactivity to the glycoprotein antigen which is provided pre-coated in a 96-Well Microplate. The antigen contains the amino acid sequence 510–690 of uniprot #Q8JSZ3. In brief, sera were diluted 1:5 or 1:10 in PBS in 96 well plates, incubated at 37°C for 30 min and processed with HRP conjugate reagent and TMB Substrates according to the manufacturer’s protocol.

### Indirect Immunofluorescence (IFA) for Screening of Antibodies to Hantaviruses

Vero E6 cells were purchased from the American Type Culture Collection. Vero E6 cells were grown in complete minimal essential media (c-MEM) (Corning, NY, USA) which included 10% FBS (Gibco, Waltham, MA, USA), 5 mM penicillin/streptomycin (Gibco), and **l**-glutamine (Gibco). Cells were incubated at 37°C with 5% CO**_2_**. DOBV and PUUV were provided by Dr. Connie Schmaljohn (USAMRIID, Frederick, MD, USA). Cells were infected with 0.1 MOI of PUUV or DOBV and grown for 7 days. Virus-infected cells were harvested, aliquoted and grown overnight on 10 spot microscope slides (Fisher) and fixed in acetone as previously described (Jonsson et al., 2010a). To identify sera with antibody to hantaviral antigens, we first screened a 1:10 dilution of sera in PBS. The presence of antigen-linked antibody was detected by a second incubation with Goat anti-Human IgG (H+L) Cross-Adsorbed Secondary Antibody, Alexa Fluor 488 (Thermo Fisher). Samples were read for the presence of perinuclear specific staining using a fluorescent microscope. For sera that were positive for antibodies, we further assessed its reciprocal titer with two-fold dilutions of sera from 1:32 to 1:2048. Slides without virus were prepared and used as a negative control in the evaluation of the IFA slides along with a positive control sera from rodents. In addition, on each slide, well for a negative (no sera) control.

### Statistical Analysis

The seroprevalence of the two hantaviruses and CCHFV is coded as 1 (positive) or 0 (negative). To analyze the questionnaire data for risk, we excluded 36 subjects that were **“**not tested**”** for CCHFV IgG antibody. For exposure self-reporting, responses of **“**once per life**”** and **“**every year**”** are coded as low, and **“**every month,**” “**every week,**”** and **“**every day**”** are coded as high. The positivity of seroprevalence was modeled with Firth**’**s logistic regression with each of the eleven demographic variables as a predictor. If a predictor has three or above levels, the first level is treated as reference, the other levels are compared with the reference level, the p value on the reference level is for testing the null of all odds ratios equal to one in the ANOVA.

### Geographical Analyses

The unique latitude/longitude combinations in the data set were extracted and summary statistics were calculated to determine the number of samples collected at each location and the number of positive and negative results for each virus. This aggregated data set formed the basis for subsequent analysis. Using QGIS (v3.03), the weighted mean center of sampled locations was calculated for each virus. To determine differences in the distributions of positive and negative results, the total number of positive tests and the total number of negative results at each location were selected as the weighting metrics. The unweighted mean center was not calculated explicitly. The SD (Standard Distance) measures were calculated using the **“**standard distances**”** plugin for QGIS. Both a weighted and unweighted metric were calculated for each virus. Total counts for seropositive and seronegative samples were used as weights. The standard deviational ellipse (SDEs) were calculated using the **“**standard deviation ellipse**”** plugin for QGIS. Weighted and unweighted metrics were calculated. The **“**Yuill**”** method with square root 2 and degrees of freedom corrections was applied. These corrections ensure that if there is a directionally unbiased, random distribution of points the radius of the SDE will be equivalent to the SD. A discrete spatial Bernoulli model was used with SatScan to identify high-value clusters of antibody-positive samples. The Bernoulli model identifies clusters where the variable of interest can be represented in binary form (1/0, cases or non-cases). This allows us to identify clusters when the number of samples tested is not uniform across the study area. In this application, the variable is the number of samples that are positive or negative for each pathogen. The scan statistic settings were adjusted to allow for multiple clusters with no geographic overlap (a location cannot be included in more than one cluster and will be preferentially included in the more likely cluster). The maximum spatial cluster size was set to no more than 50 percent of the population at risk.

## Results and Discussion

### The Study

**Figure 1:**
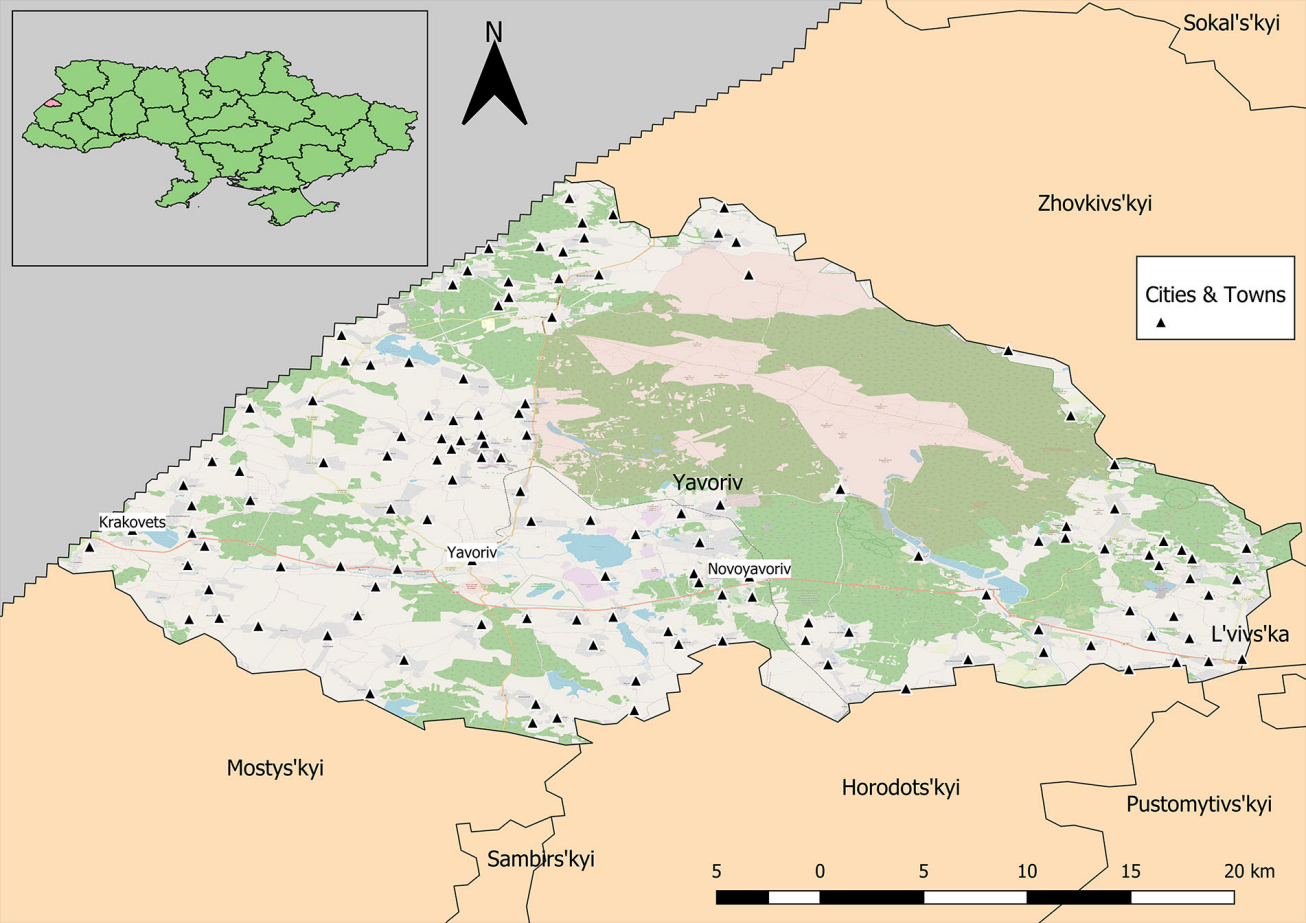
Yavoriv rayon in Lviv Oblast, Ukraine. Cities and towns are distributed across the rayon with the exception of the protected military and natural areas in the northeastern region.

The study region was located within Lviv Oblast, which lies in western Ukraine and shares international boundaries with Poland to the west (**Figure 1**). The oblast encompasses approximately 20,100 km^2^ with more than one million inhabitants. The study was a qualitative serosurvey, which utilized convenience sampling from blood donors and potentially non-infected hospital patients. Sera samples were collected from healthy volunteers ***via*** cooperating hospitals, which resulted in the creation of a serum bank. Two primary hospitals, Novoyavorivsk and Yavoriv, were engaged in the study; both have blood donation centers attached to them.

**Figure 2:**
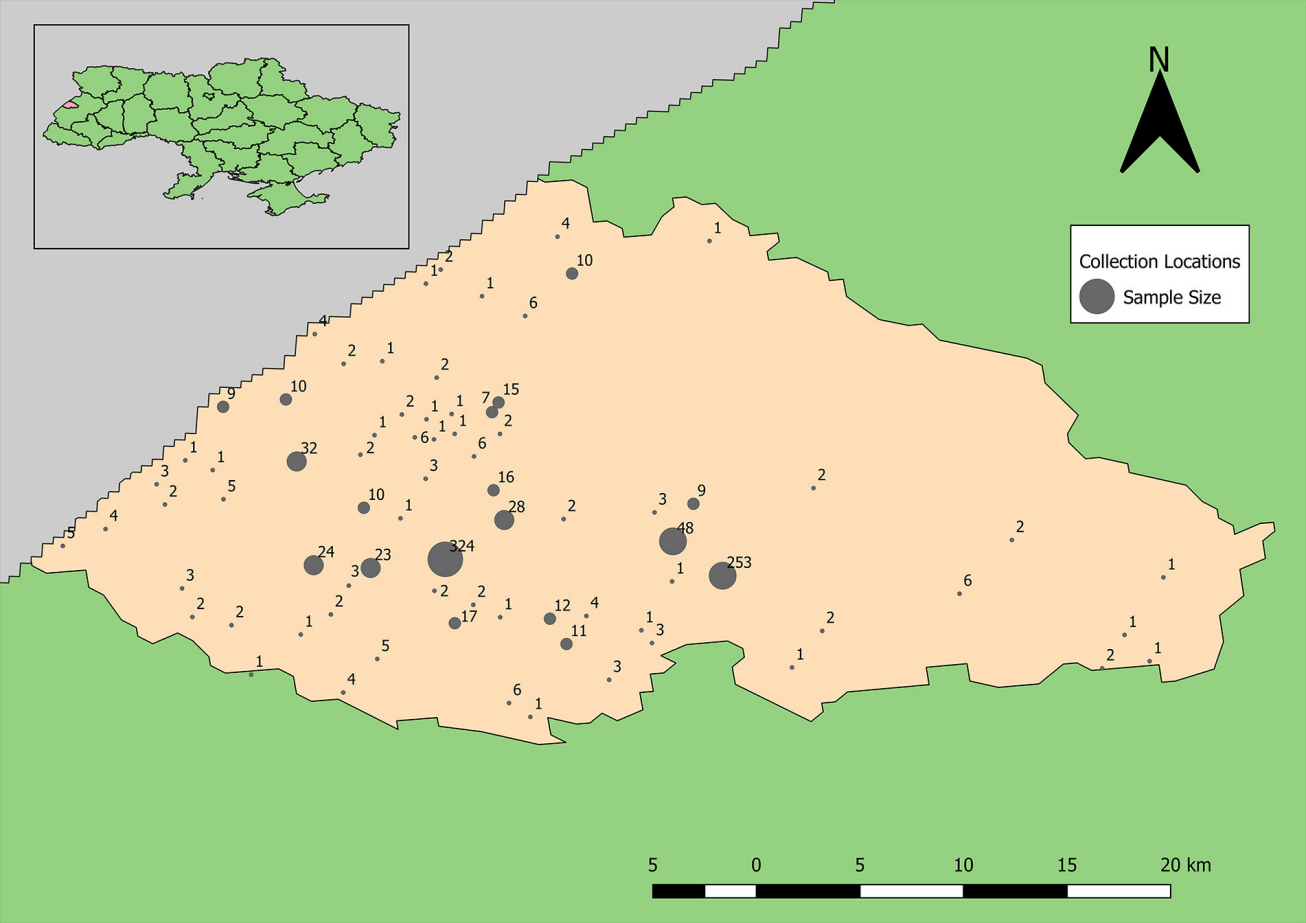
Distribution of human sera samples collected and tested for antibodies to Old World hantaviruses and CCHFV by unique location (towns and villages of subject’s residence).

Demographic analyses of 996 completed surveys of the total 1,002 donor records indicated that the average age was 41.5 years of age and 60.4% of the volunteers were female; 300 volunteers resided in Yavoriv, approximately 150 were from Novoyavorivsk, and the remainder were from miscellaneous towns (**Figure 2**). Among the 996 volunteers, 87.6% reported that their city of residence was their permanent residence, 56.4% classified their residence as urban, and 93% resided in the same location their entire life.

### CCHFV

We used an indirect immunofluorescence assay (IFA) to screen 966 human sera for the presence or absence of antibodies cross-reactive with PUUV or HTNV viruses grown in Vero E6 cells (Jonsson et al., 2010b). Using the HTNV antigen IFA slides, a total of 14 samples were positive. Two samples tested positive in titration at 1:10 (level of screening), five were positive in titration at 1:32, and seven were positive at 1:16 (**Table 1**). Using PUUV antigen IFA slides, sera were screened using dilutions of 1:10, resulting in only one sample positive by IFA (**Table 1**).

**Table 1:** Summary of the number of healthy subjects with antibodies to PUUV or HTNV antigens by IFA and CCHFV by ELISA, with reciprocal titers cited.

Reciprocal Titers					
Species	1:5	1:10	1:16	1:32	Total
*PUUV*				1	1
*HTNV*		2	7	5	14
*CCHFV*	14	2			16

Of the 966 samples, sera were diluted 1:5 and examined using a commercial ELISA. Samples that were positive were examined again at 1:10 in a second IgG ELISA. Of these 16 samples (1.7%) were confirmed positive for prior infection with CCHFV (**Table 1**).

### Risk Factors Assessment for Association with Seropositive Hantaviruses or CCHFV

Logistical regression demonstrated no association of basic demographic factors (age, gender, type of residence) and main expected exposures (contact with rodents, visiting forest or fields) and presence of antibodies to hantaviruses; while, the odds for the presence of antibodies to hantaviruses in subjects with every year contact with ticks was 11.55 times of those without contact (p = 0.04) (**Table 2**). This was also true for most of the same factors and presence of antibodies to CCHFV; however, being in the forest and cleaning in and around the house more often demonstrated negative association with being seropositive for CCHFV (p = 0.025, 0.048, respectively) (**Table 3**).

**Table 2:** Risk factors (basic demographics and main exposures) and their association with presence of antibodies to hantaviruses.

Predictor	Levels	Number of subjects	Number of positive results	Odds Ratio	95% CI	*P*-value
Hospital	Novoiavorivsk Rayon Hospital	336	6	1.0000	–	
	Yavoriv Central Rayon Hospital	630	9	0.7971	0.2813–2.2588	0.66958147
Gender	female	611	10	1.0000	–	
	male	355	5	0.8586	0.2911–2.5323	0.78229868
Age group	(<=28)	182	5	1.0000	–	0.41128098
	(29–38)	234	4	0.6157	0.1629–2.3263	0.47448348
	(39–48)	261	3	0.4116	0.0971–1.7445	0.22830605
	(49–58)	199	1	0.1788	0.0207–1.5451	0.11767731
	(59–68)	67	2	1.0892	0.2062–5.7533	0.91982265
	(69 above)	21	0	0.0000	0 to Inf	0.99159203
Settlement type	town	554	7	1.0000	–	
	Village	410	8	1.5551	0.5593–4.3235	0.39737256
Self-reported rural or urban	rural	409	8	1.0000	–	
	urban	556	7	0.6391	0.2299–1.7769	0.39084082
Past medical history: pneumonia	no	858	11	1.0000	–	
	yes	108	4	2.9615	0.9262–9.4695	0.06714095
Exposure: contact with rodents	NO	885	14	1.0000	–	0.7524809
	Low	63	1	1.0035	0.1298–7.7565	0.99736163
	High	18	0	0.0000	0 to Inf	0.98850422
Exposure: going to forest	NO	78	1	1.0000	–	0.35627003
	Low	541	11	1.5981	0.2035–12.552	0.65571586
	High	347	3	0.6715	0.0689–6.5432	0.73172098
Exposure: going to field	NO	80	1	1.0000	–	0.68246152
	Low	227	5	1.7793	0.2047–15.465	0.60147352
	High	659	9	1.0938	0.1368–8.7484	0.93261235
Exposure: cleaning in or around house	NO	205	4	1.0000	–	0.27861704
	Low	75	0	0.0000	0 to Inf	0.98994512
	High	686	11	0.8189	0.258–2.5996	0.73459958
Exposure: contact with ticks	NO	763	11	1	–	0.1191
	Once per life	192	3	1.209	0.305–3.691	0.7623
	Every year	9	1	8.5455	11.547	1.163–58.362

**Table 3:** Risk factors (basic demographics and main exposures) and their association with presence of antibodies to CCHFV.

Predictor	Levels	Number of subjects	Number of positive results	Odds Ratio	95% CI	*P*-value
Hospital	Novoiavorivsk Rayon Hospital	336	10	1.000000e+00	–	
	Yavoriv Central Rayon Hospital	630	6	3.135000e−01	0.1129–0.8701	0.02594173
Gender	female	611	13	1.000000e+00	–	
	male	355	3	3.920000e−01	0.111–1.3853	0.14595199
Age group	(<=28 years)	182	0	1.000000e+00	–	0.1658475
	(29–38 years)	234	5	1.865798e+07	0 to Inf	0.98983639
	(39–48 years)	261	6	2.010672e+07	0 to Inf	0.989791
	(49–58 years)	199	4	1.752893e+07	0 to Inf	0.98987428
	(59–68 years)	67	1	1.294751e+07	0 to Inf	0.99005819
	(69 years above)	21	0	1.000000e+00	0 to Inf	1
Settlement type	Town	554	8	1.000000e+00	–	
	Village	410	8	1.358200e+00	0.5055–3.6495	0.54377659
Self-reported rural or urban	Rural	409	9	1.000000e+00	–	
	Urban	556	7	5.667000e−01	0.2093–1.5344	0.26377186
Past medical history: pneumonia	No	858	15	1.000000e+00	–	
	Yes	108	1	5.252000e−01	0.0687–4.0161	0.53498221
Exposure: contact with rodents	No	885	15	1.000000e+00	–	0.73669307
	Low	63	1	9.355000e−01	0.1216–7.1988	0.94892491
	High	18	0	0.000000e+00	0 to Inf	0.98844421
Exposure: going to forest	Exposure: going to forest	NO	78	4	1	–
	Low	Low	541	10	0.327	0.111–1.129
	**High**	**High**	347	2	0.12	0.021–0.551
Exposure: going to field	Exposure: going to field	NO	80	2	1	–
	Low	Low	227	5	0.776	0.183–4.388
	High	High	659	9	0.459	0.127–2.43
Exposure: cleaning in or around house	Exposure: cleaning in or around house	NO	205	7	1	–
	Low	Low	75	2	0.9	0.165–3.465
	**High`**	**High**	686	7	0.292	0.103–0.831
Exposure: contact with ticks	NO	763	15	1.000000e+00	–	0.24710644
	Once per life	192	1	2.611000e−01	0.0343–1.9889	0.19487567
	Every year	9	0	0.000000e+00	0 to Inf	0.99173727

### Spatial Distribution of Positive Results for Antibodies to Hantaviruses and CCHFV

**Figure 3:**
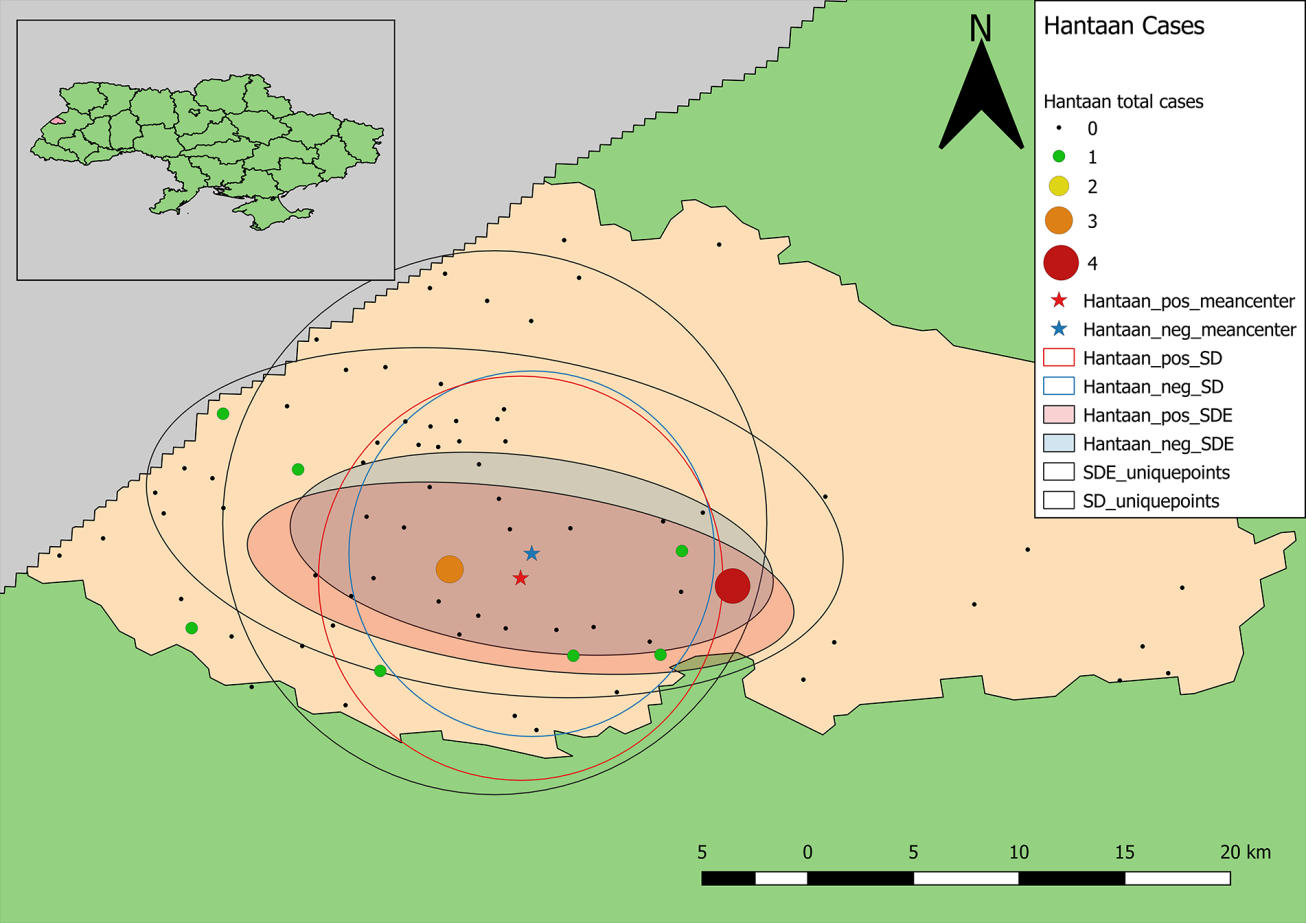
Descriptive statistics showing differences in the distribution of sample locations, and positive and negative results for antibodies to Old World hantaviruses (blue representing negative test results, while red represents the distribution of positive cases). Centering of distributional measures to the southwest indicates slight spatial bias in sample locations. Smaller radii for positive and negative weighted measures indicate tighter distribution of these values within the distribution of sample locations, overall.

Calculation of unweighted SD and SDE indicate that within the study area, the distribution of samples is biased to the south and west (**Figures 3**, **5**). The SD and SDE are centered to the southwest indicating greater density of samples in the region. This is largely due to the presence of the large forested area in the northeastern region of the rayon that is devoid of settlements (**Figure 1**). However, the large radius of the SD of sample locations relative to the study area indicates moderately good coverage (especially in the southwest). The major axis of the SDE lies in line with the dominant shape of the rayon (east-west).

Samples positive for antibodies to hantaviruses were dispersed across the southwestern and south-central part of the rayon (**Figure 3**). The total number of positive samples from a given location ranged from 1 to 4 with only two locations reflecting three or more positive samples. These were locations with large numbers of recruited samples where the hospitals were located (**Figure 2**).

The mean centers of total positive and negative tests for antibodies to hantaviruses are shifted southward relative to the distribution of sample locations. In addition, the mean center of positive tests is shifted even further south than that of negative cases. The radius of the SD of positives is slightly larger than that of negatives indicating a broader distribution of positive cases around the weighted mean center. The SDE of positive and negative tests reflect similar east-west directionality although the difference between major and minor axes in the positive-weighted SDE indicates this directionality is slightly stronger (there is greater east-west variation in the number of positive cases).

**Figure 4:**
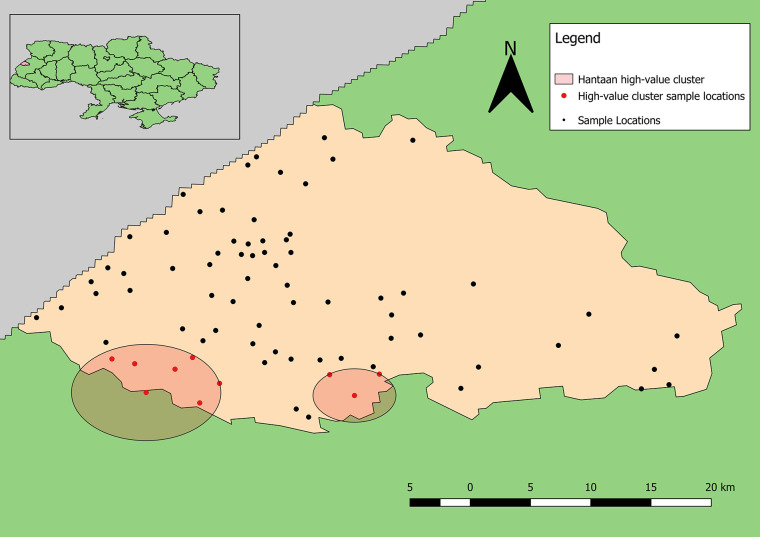
Clusters of high-value results for antibodies to Old World hantaviruses. Both clusters are statistically non-significant (p >0.05).

The point pattern analysis identified two statistically non-significant clusters of samples positive for antibodies to hantaviruses (p > 0.05). Both clusters contained several locations that included a low number of positive samples, but also fewer negative results than expected. The primary cluster, centered near Shchyhli, contained two locations that reported positive samples for HTN as well as several that did not (**Figure 4**). The secondary cluster, near Hlynets, also contained two locations reporting positive samples, and an additional location where no positive results were reported.

**Figure 5:**
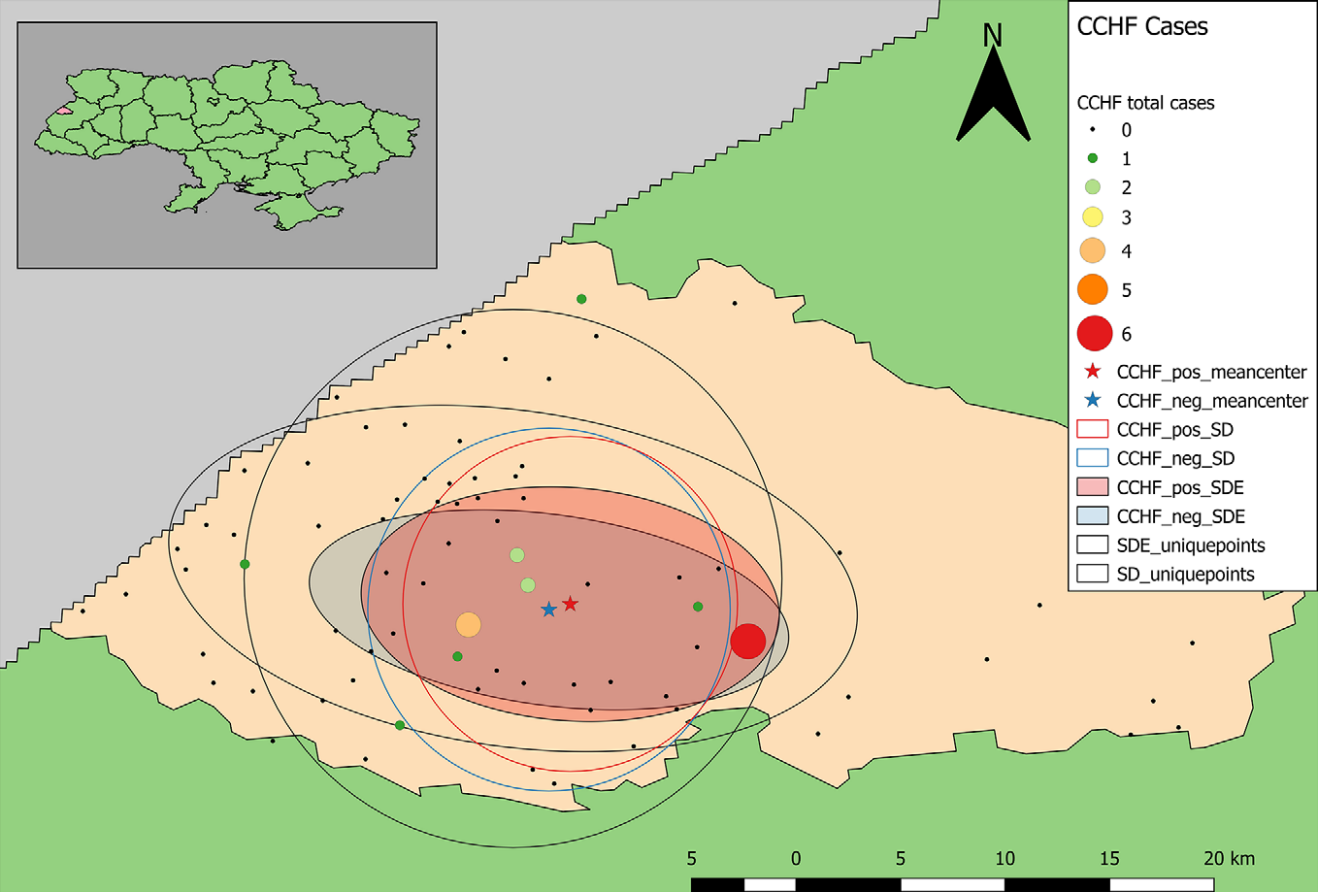
Descriptive statistics showing differences in the distribution of sample locations, and positive and negative results for antibodies to CCHFV (blue representing negative test results, while red represents the distribution of positive cases). Centering of distributional measures to the southwest indicates slight spatial bias in sample locations. Smaller radii for positive and negative weighted measures indicate tighter distribution of these values within the distribution of sample locations overall.

Samples positive for antibodies to CCHFV were largely restricted to the south-central part of the rayon (**Figure 5**). The total number of positive samples from a given location ranged from 1 to 6 with only two locations having four or more positive samples, which were the same as for samples positive for antibodies to hantaviruses. The mean centers of total positive and negative tests for antibodies to CCHFV are shifted eastward relative to the distribution of tested samples (**Figure 5**). In addition, the mean center of positive tests is shifted even further eastward than that of negative cases. The radius of the SD of positives is slightly smaller than that of negatives indicating a tendency for clustering of positive cases around the weighted mean center. The SDE of positive and negative tests reflect similar east-west directionality although the difference between major and minor axes in the positive-weighted SDE indicates this directionality was not as strong (or that north-south directionality is more significant in this case).

**Figure 6:**
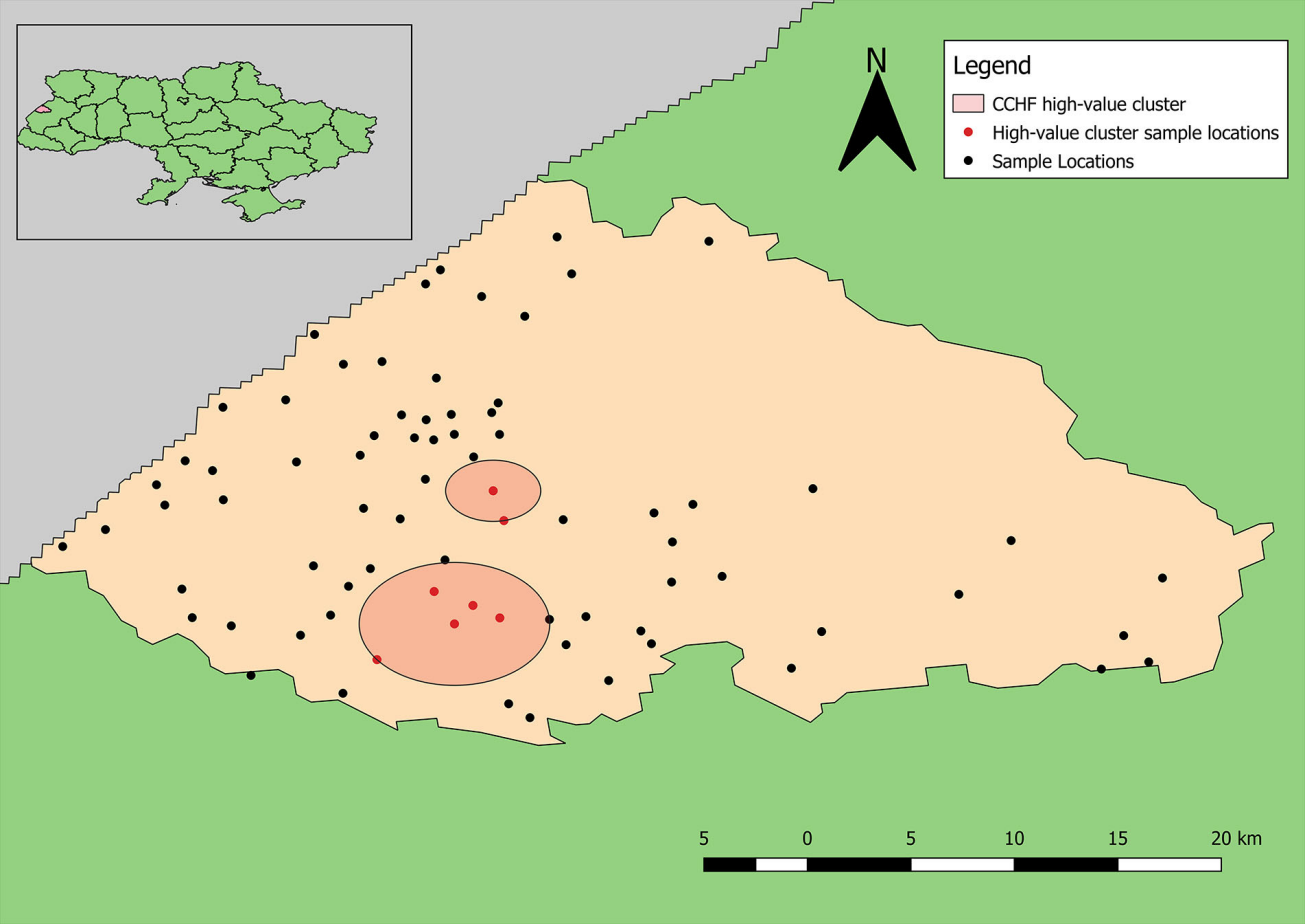
Clusters of high-value results for antibodies to CCHFV. Neither cluster was statistically significant (p > 0.05).

The point pattern analysis identified two statistically non-significant clusters of samples positive for antibodies to CCHFV (p > 0.05). Both clusters contained several locations that included a low number of positive samples, but also fewer negative results than expected. The primary cluster, near Yavoriv and Rulevo, contained two locations, both of which reported positive samples for antibody to CCHFV antigen (**Figure 6**). The secondary cluster near Staryi Yar also contained two locations reporting positive samples, and an additional three locations where no positive results were included.

## Discussion

In Ukraine, cases of HFRS have been suspected based on clinical symptoms since the first recognition of the disease during the Korean War in the 1950s. This study suggests that people in Lviv Oblast of Ukraine are exposed to hantaviruses and CCHFV. Seroprevalence of CCHF in our study was 1.7% which is comparable with several regions of Turkey and lower than had been demonstrated for Bulgaria, Hungary, Serbia, Georgia and most of Turkey’s regions (Monsalve-Arteaga et al., 2020). However, unlike Ukraine, these countries report human CCHF cases. In terms of spatial patterns, generally, there is little evidence of spatial clustering of detecting antibodies to hantaviruses and CCHFV beyond reflecting recruitment success of study participants and/or general human population distribution. However, for CCHFV, the distribution of positive antibody samples is slightly pulled towards the north and east, relative to the distribution of negative samples. This is a region of the rayon where extensive forests could maintain a CCHFV transmission cycle. A combination of smaller positive-weighted SD and smaller difference between major and minor axes for positive-weighted SDE indicate a tighter distribution of positive results (higher density of positive tests). The spatial characteristics of samples positive for antibodies to hantaviruses indicates that positive results are more broadly dispersed than negative results. The larger SD and SDE (along the major axis) for positive results compared to that of negative-weighted results and a southern shift in the weighted mean center indicate a broader distribution of occurrence in this region. Perhaps most interesting are the differences in the distributions of positive results for antibodies to CCHFV versus hantaviruses. The mean center of positive results for antibodies to CCHFV is further north and east than that of antibodies to hantaviruses. Additionally, smaller SD and reduced directional variation in positive results for antibodies to CCHFV is indicative of a more limited geographic distribution. That the CCHF pattern differs from the hantaviruses pattern suggests the distribution of positive samples is not solely a reflection of sampling patterns in participant recruitment.

The high-value clusters of detecting antibodies to CCHFV bordered two large natural/forested areas. Neither location reporting the highest numbers of positive samples for antibodies to CCHFV were included in the clusters likely because much higher total samples were collected from these locations; however, one of these locations is located directly between the reported clusters of detecting antibodies to CCHFV. This distinction shows the advantage of applying explicit statistical analyses to interpret spatial patterns. The second location reporting the highest number of positive samples is located near the border of Yavoriv National Park. Similarly, the locations reporting the highest numbers of positive samples for antibodies to hantaviruses also border these natural areas, although the identified high-value clusters are not as close.

The absence of statistically significant spatial clusters has two, non-exclusive explanations. First, the relative low prevalence rates (<5%) for detecting antibodies to both CCHFV and hantaviruses means that there are relatively few positive locations from which to generate evidence of “clustering.” Thus, sites with fewer than 20 individuals tested might be in environmentally high-risk zones but by chance, alone, none of the participants were serologically positive. A second interpretation is that the entire rayon is a high-risk zone and the samples tested simply reflect the background level of risk for the region. This second explanation appears less likely as the spatial clustering analysis, though finding high risk clusters to not be statistically significant, did not incorporate all the sites where the largest numbers of positive individuals were sampled.

Homogeneity of the study population in terms of the level of risk may also be considered an explanation of no association found between being positive for antibodies to CCHFV and hantaviruses and demographic factors and exposure history. For example, while more than half of the study subjects reported living in an “urban” environment, this was more due to formal status of town versus village than a reflection of living conditions and exposures. Unexpected “protective” effect of being in a forest and cleaning in and around a house (against exposure to CCHF) might be due to some confounder or gaps in interview study subjects (doubtful combinations of answers were seen; e.g., working in forestry and being in a forest only once a year, living in rural area and not seeing rodents, etc.). This could also influence absence of association with other anamnestic factors collected based on participants.

Ultimately, the spatial and statistical analyses depend on the accuracy of diagnostic assays themselves. The spatial and statistical analyses do not account for issues related to sensitivity or specificity of laboratory tests. Changes in the thresholds applied to define a positive sample or altering protocols also might impact the patterns reported here.

## Data Availability Statement

All data sets presented in this study are included in the article/**Supplementary Material**.

## Ethics Statement

The studies involving human participants were reviewed and approved by The US Defense Threat Reduction Agency (DTRA). Human Research Oversight Board (HROB) reviewed and approved this research study, which was conducted in compliance with U.S. Army Regulation AR 70-25. The protocol, informed consent document, and US Army Medical Research Institute of Infectious Diseases (USAMRIID) Institutional Review Board (IRB) approval complied with 32 CFR 219, DoD 3216.2, DTRA Directive 3216.01, and DTRA Instruction 3216.02. The tracking and study numbers were CT-2009-07-Other and FY 08-32, respectively. The patients/participants provided their written informed consent to participate in this study.

## Author Contributions

CJ and IL contributed conception and design of the study. MT, BS-H, EW, AS, and OZ performed wet lab benchwork. ON, IB, WK, GG, and XC contributed to computational and geographic analyses. All authors contributed to the article and approved the submitted version.

## Funding

This work was funded by DTRA through the Biological Threat Reduction Program in Ukraine. The contents of this publication are the responsibility of the author and do not necessarily reflect the views of DTRA or the United States Government.

## Conflict of Interest

ON was employed by Black and Veatch.

The remaining authors declare that the research was conducted in the absence of any commercial or financial relationships that could be construed as a potential conflict of interest.

The handling editor declared a past advisory role with one of the authors [CJ].
