# Supplementary material for: Seroprevalence of Old World Hantaviruses and Crimean Congo Hemorrhagic Fever Viruses in Human Populations in Northwestern Ukraine
Source: Front Cell Infect Microbiol. 2020 Oct 22;10:589464. doi: 10.3389/fcimb.2020.589464 (PMC7642871; doi:10.3389/fcimb.2020.589464)
Supplement: Supplementary file 1 [file DataSheet_1.docx]

SEROPREVALENCE QUESTIONNAIRE

**Date of Interview: ____/______/______ VIN: __________________________**

**Household ID: _________________**

| **Eligibility Criteria** | | |
| --- | --- | --- |
| Does the volunteer live in a selected household? | Yes | No |
|  |  |  |
| Is the volunteer at least 18 years old? Age: __________ | Yes | No |
|  |  |  |

*I would like to ask you a few questions about your background.*

**Demographic Information**

**What is your name?** __________________ ____________________ _________________

*Last First Patronymic*

**What is your DOB?** ____/_____/____

*dd/mm/yyyy*

**Gender:** M F

**What is your address?** __________________________ City/Village? ___________________

**Is this your permanent residence?**  Current Permanent  Unknown

**What is your ethnicity? (Specify) _________________________**

**What is your occupation?** **(Specify)** __________________________

**How many years of formal education have you received?** __________

**How many persons currently live in your household?** ________ person(s)

**How many rooms are in your household?** _____________ rooms

**Health**

***Presence of Mild/Generalized Illness***

**1. How many times have you had a fever in the last 5 years?**

Never  1 Time  2 Times  3 Times  4 Times  5 or More Times

**Which season did the fever(s) usually occur in?**

Spring Summer Fall Winter Don’t remember  All different seasons

**2. How many times have you had a stomach illness (nausea, vomiting, and/or diarrhea) in the last 5 years?**

Never  One Time  Two Times  Three Times  Four Times  Five or More Times

**Which season did the stomach illness(s) usually occur in?**

Spring Summer Fall Winter Don’t remember  All different seasons

**3. How many times have you had a rash in the last 5 years?**

Never  One Time  Two Times  Three Times  Four Times  Five or More Times

**Which season did the rash usually occur?**

Spring Summer Fall Winter Don’t remember  All different seasons

**4. How many times have you noticed yellowing of your skin or eyes in the last 5 years?**

Never  One Time  Two Times  Three Times  Four Times  Five or More Times

**Which season did the yellowing of your skin or eyes usually occur?**

Spring Summer Fall Winter Don’t remember  All different seasons

***Presence of Severe Illness***

**5. Have you ever had a fever that lasted more than a week?** YesNo

**If yes, how many times?** ________

**Which season did it usually occur in?**

Spring Summer Fall Winter Don’t remember  All different seasons

**6. Outside of injury, have you ever had any unusual bleeding?** YesNo

**If yes, how many times?** ________

**Which season did it usually occur in?**

Spring Summer Fall Winter Don’t remember  All different seasons

**7. Have you ever been diagnosed with any of the following?:**

**If “Yes” please indicate approximately how long ago your diagnosis was**

**Yes No 1Month 1 Year 5 Years 10 Years 20 Years > 20 Years**

**Meningitis**

**Encephalitis**

**Kidney Failure**

**Pneumonia**

**Brucellosis**

**Hemorrhagic fever**

**Tuberculosis**

**Lyme Disease**

**Typhus**

**Cholera**

**Polio**

**Typhoid**

**Spotted Fever**

**Epidemiology**

**8.Have you been in frequent contact with any of the following animals (check all that apply)?**

| **None** | **Swine** | **Pigeons** | **Dogs** |
| --- | --- | --- | --- |
| **Sheep** | **Donkeys** | **Ducks** | **Bats** |
| **Goats** | **Horses** | **Other Birds** | **Rodents** |
| **Cattle** | **Chickens** | **Cats** | **Rabbits** |

**Other_________________________________________________________________________**

**9. Do you *regularly* conduct any of the following activities (check all that apply):**

| **Handle animal hides** | **Go Hunting** | **Contact bodies of water (rivers, lakes, ponds, etc)?** |
| --- | --- | --- |
| **Shearing sheep** | **Go Fishing** | **Sweep inside or around your home?** |
| **Milking Animals** | **Go to wooded areas** | **Directly contact sick livestock?** |
| **Slaughtering Animals** | **Go to fields or meadows** |  |
| **Sleep outside** | **Assist in animal birth?** |  |

**10. Do herds of animals pass by your home regularly?** Yes No

**11. Do you consume milk or dairy products?** Yes No

**If you answered “yes” please answer the following:**

**How often do you consume milk or dairy products?**

Daily  Weekly  Monthly  Yearly  A few times in my life

**Are the dairy products you consume**  Pasteurized  Unpasteurized  Both

**How often do you consume unpasteurized dairy products?**

Never  Daily  Weekly  Monthly  Yearly  A few times in my life

**Where do you typically obtain dairy products?**

Commercial Market/Store  Open Market/Bazaar  Make at home  Other_________

**12. Do you consume meat or meat products?**  Yes  No

**If you answered “yes” please answer the following:**

**Have you consumed meat that was red on the inside or raw?**  Yes  No

**If yes, how often do you consume meat that was red on the inside, undercooked, or raw?**

Daily  Weekly  Monthly  Yearly  A few times in my life

**Where do you typically obtain meat products?**

Commercial Market/Store  Open Market/Bazaar  Slaughter at home  Other__________

**13. How often have you found a tick on you?**

Never  Daily  Weekly  Monthly  Yearly  A few times in my life

**14. How often have you found a tick on your pets or livestock?**

Never  Daily  Weekly  Monthly  Yearly  A few times in my life

**15. How often do you notice mosquitoes?**

Never  Daily  Weekly  Monthly  Yearly  A few times in my life

HealthCare Access

16. Where do you seek healthcare when you are mild/moderately ill? ________

What is the approximate distance you travel to receive this care? ___________

18. Where do you seek healthcare when you are extremely ill? __________

What is the approximate distance you travel to receive care? _________

19. How often have you accessed healthcare in the past 5 years?

Never  A few times in my life  Daily  Weekly  Monthly  Yearly

20. How many times in the last year have you obtained antibiotics? ________

Where do you get your package of antibiotics? __________

21. If possible, name three of the most common antibiotics you use when you are sick:

____________________________________________________________________

____________________________________________________________________

____________________________________________________________________
